# Recommendations for initial diabetic retinopathy screening of diabetic patients using large language model-based artificial intelligence in real-life case scenarios

**DOI:** 10.1186/s40942-024-00533-9

**Published:** 2024-01-24

**Authors:** Nikhil Gopalakrishnan, Aishwarya Joshi, Jay Chhablani, Naresh Kumar Yadav, Nikitha Gurram Reddy, Padmaja Kumari Rani, Ram Snehith Pulipaka, Rohit Shetty, Shivani Sinha, Vishma Prabhu, Ramesh Venkatesh

**Affiliations:** 1 Department of Retina and Vitreous, Narayana Nethralaya Eye Hospital, #121/C, 1st R Block, Chord Road, Rajaji Nagar, Bengaluru, Karnataka 560010 India; 2 Medical Retina and Vitreoretinal Surgery, University of Pittsburgh School of Medicine, 203 Lothrop Street, Suite 800, Pittsburg, PA 15213 USA; 3 Anant Bajaj Retina Institute, L V Prasad Eye Institute, Kallam Anji Reddy Campus, Hyderabad, Telangana 500034 India; 4 Prime Retina Eye Care Center, 3-6-106/1, Street Number 19, Opposite to Vijaya Diagnostic Centre, Himayatnagar, Hyderabad, Telangana 500029 India; 5 Department of Cornea and Refractive Services, Narayana Nethralaya Eye Hospital, #121/C, 1st R Block, Chord Road, Rajaji Nagar, Bengaluru, Karnataka 560010 India; 6 Department of Vitreo-Retina, Regional Institute of Ophthalmology, Indira Gandhi Institute of Medical Sciences, Sheikhpura, Patna, Bihar 800014 India

**Keywords:** New cases, Diabetes, Screening, Diabetic retinopathy, Artificial intelligence

## Abstract

**Purpose:**

To study the role of artificial intelligence (AI) to identify key risk factors for diabetic retinopathy (DR) screening and develop recommendations based on clinician and large language model (LLM) based AI platform opinions for newly detected diabetes mellitus (DM) cases.

**Methods:**

Five clinicians and three AI applications were given 20 AI-generated hypothetical case scenarios to assess DR screening timing. We calculated inter-rater agreements between clinicians, AI-platforms, and the “majority clinician response” (defined as the maximum number of identical responses provided by the clinicians) and “majority AI-platform” (defined as the maximum number of identical responses among the 3 distinct AI). Scoring was used to identify risk factors of different severity. Three, two, and one points were given to risk factors requiring screening immediately, within a year, and within five years, respectively. After calculating a cumulative screening score, categories were assigned.

**Results:**

Clinicians, AI platforms, and the “majority clinician response” and “majority AI response” had fair inter-rater reliability (k value: 0.21–0.40). Uncontrolled DM and systemic co-morbidities required immediate screening, while family history of DM and a co-existing pregnancy required screening within a year. The absence of these risk factors required screening within 5 years of DM diagnosis. Screening scores in this study were between 0 and 10. Cases with screening scores of 0–2 needed screening within 5 years, 3–5 within 1 year, and 6–12 immediately.

**Conclusion:**

Based on the findings of this study, AI could play a critical role in DR screening of newly diagnosed DM patients by developing a novel DR screening score. Future studies would be required to validate the DR screening score before it could be used as a reference in real-life clinical situations.

**Clinical trial registration:**

Not applicable.

**Supplementary Information:**

The online version contains supplementary material available at 10.1186/s40942-024-00533-9.

## Introduction

Diabetes mellitus (DM) is a worldwide epidemic that causes a variety of complications in the human body [1]. DM-related vascular complications usually develop after a few years, and many individuals, especially those from middle- and low-income countries, do not have annual DM diagnosis check, leaving a large group undiagnosed [2]. Diabetic retinopathy (DR) is one of the many serious eye-related complications of DM [3, 4]. DM patients are screened for DR to identify and treat sight-threatening DR (proliferative DR and/or diabetic macular edema) and to recommend follow-up for those without DR or non-proliferative DR without diabetic macular edema [5, 6]. Several population-based studies have found a disproportionate prevalence of DR around the world, with countries in the Middle East, North Africa, and the Western Pacific having the highest prevalence and countries in the South and Central America having the lowest [7]. In comparison to Western countries, the prevalence of DR in India is low, with estimates ranging from 5 to 16%. The most recent publication from the SMART India Study group found a national prevalence of 12.5% for DR and 4.0% for sight-threatening DR [6, 8]. This is despite the fact that India has the world’s second-highest number of people with DM [9]. The main reason cited for this uneven distribution of DR cases worldwide is the different screening strategies followed by different countries [10]. Furthermore, the personnel conducting the DR screening, the DR classification used, and the presence of other systemic co-morbidities all have an impact on determining the exact prevalence of DR [11, 12].

Other than the retina specialists, the initial DR screening for newly detected cases of DM is usually carried out by other ophthalmologists, and non-ophthalmologists such as optometrists and diabetologists using dilated fundoscopy or teleophthalmology tools such as mydriatic and non-mydriatic fundus cameras [10]. There are differences in the initial timing for DR screening even among ophthalmologists. These distinctions are primarily due to the area (urban/rural) in which they practice, the type of institution to which they are affiliated, and the types of patients they screen [13]. Diabetes patients have a large urban-rural divide, which hinders disease understanding and prevents routine screening as per established protocols. A streamlined strategy for initial DR screening would assist medical screening staff and patients in determining when to be screened.

AI has been debated for its potential benefits and drawbacks in medicine, including ophthalmology. Several studies have used fundus photos and deep machine learning AI for DR screening [14–16]. However, there are concerns about data acquisition, bias in data, bias in identifying ground truth, difficulty comparing different algorithms, challenges in machine learning, its application in different groups of people, and human barriers to AI adoption in health care [17]. A large language model (LLM) or natural language processing algorithm is a form of generative AI that uses massive data sets to understand, summarize, generate, and predict new text-based content [18]. Many such open source LLM-based generative AI algorithms are currently freely and easily available, including OpenAI’s ChatGPT3.5v and ChatGPT4.0v Google’s BARD, Microsoft’s Bing AI, and others [19]. Most researchers and clinicians believe that AIs based on LLM could help reduce physician burden if integrated into the electronic health record [20].

In countries with a large population and a low ophthalmologist-to-patient ratio, retina specialists screening all newly detected diabetic patients with dilated fundus examination would be demanding, not enhance the yield of DR cases, and reduce the ophthalmologist’s time for other patients [21]. We believe that AI can simplify screening recommendations for non-ophthalmologists like medical internists and DM specialists, as well as general ophthalmologists, to guide newly diagnosed DM patients to retina specialists for DR screening. We found no literature on LLM-based AI for DR screening in newly diagnosed DM.

Thus, the primary goal of this study was to investigate the role of AI in establishing a streamlined method for determining the appropriate timing of initial screening for DR with the help of ophthalmologists and various AI platforms.

## Methods

This was a prospectively conducted questionnaire-based study. The study commenced by requesting ChatGPT 3.5v (OpenAI, San Francisco, CA, USA) to generate 20 hypothetical clinical case scenarios pertaining to DM and the necessity and timing for an initial dilated fundus examination conducted by an expert retina specialist. This was accomplished by utilizing various combinations and permutations of the specified keywords, including age, gender, duration, type and control of DM, obesity, kidney disease, blood pressure, cholesterol, tobacco use, pregnancy status, and family history of DM (Supplement 1).

The clinical case scenarios were subsequently distributed to a group of five retina specialists/clinicians who possessed at least over five years of clinical experience in the field of retina and DR screening. These retina specialists have professional experience in various organizational settings, including government hospitals, independent private practices, tertiary eye care hospitals serving both free and paying patients, and tertiary eye care corporate hospitals exclusively serving paying patients. The responses provided by the clinicians to the clinical case scenarios were collected using a 3-point multiple-choice format, where each clinician was required to select only one response that best addressed the appropriate timing for DR screening in each clinical case scenario. The three response options were whether the DR screening should be done immediately, within one year, or within five years. The ‘majority clinician response’ for each specific case scenario was determined by identifying the maximum number of identical responses provided by the clinicians.

**Fig. 1:**
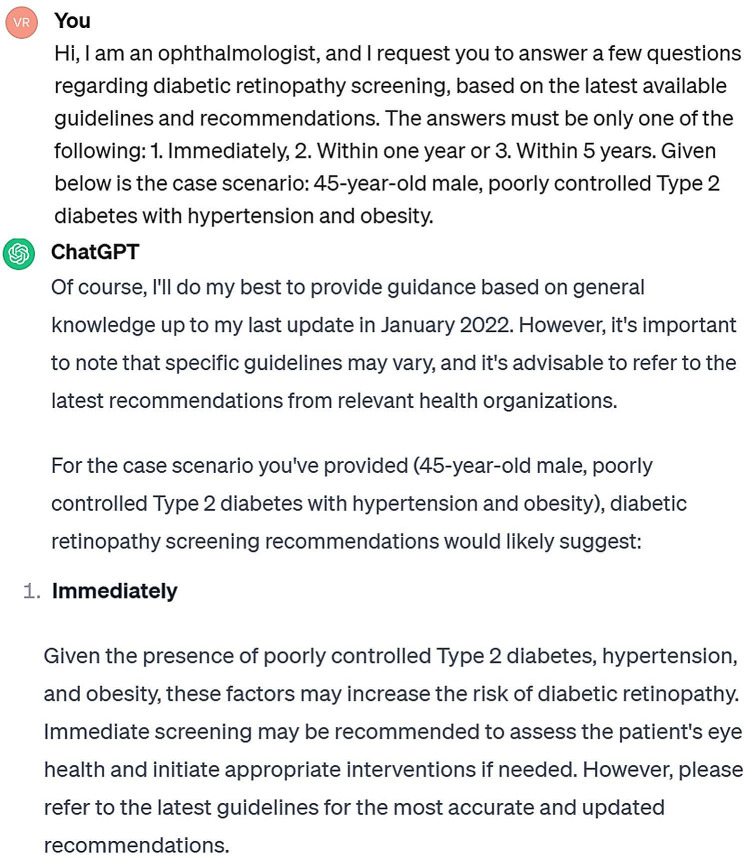
Prompt applied to request an opinion from the ChatGPT3.5v AI platform, accompanied with the AI’s response to a specific scenario from the study

Subsequently, an exact same set of clinical case scenarios with options was presented to various important AI platforms, including ChatGPT 3.5v, ChatGPT 4.0v, and Bing AI. ChatGPT 3.5v, ChatGPT 4.0v, and Bing AI were last trained in January 2022, April 2023, and somewhere in 2021, respectively. The text was entered into various AI platforms, with a specific request to provide the most appropriate single response for each clinical case scenario. The responses were generated using the same set of multiple-choice options that were presented to the clinician. The query was formulated in a manner that implies the clinician is asking about the most suitable time for a patient’s first dilated retinal examination, rather than the patient seeking advice from the AI on when to schedule a visit to a retina specialist for a dilated fundus examination. The AI did not receive any feedback after each case scenario, and the case descriptions were inputted in a sequential manner without initiating a new chat session. Figure 1 depicts the prompt used to generate an opinion as well as the AI response. The formal responses for each case scenario were documented based on the outcomes generated by various AI platforms. The determination of the ‘majority AI response’ for each specific case scenario was made by identifying the highest count of identical responses among the three distinct AI platforms. For each individual clinical case scenario, the ‘majority clinician response’ was compared to the ‘majority AI response’ for agreement.

Based on the responses obtained from the clinicians and different AI platforms, a consensus was reached on the ‘most common’ response for each individual case scenario for determining the optimal timing for initial screening for diabetic patients with dilated fundus examination by a retina specialist. The determination of the ‘majority response’ involved identifying the response with the highest frequency among the clinicians and AI platforms. Specifically, a maximum of eight responses were considered, consisting of five from the clinicians and three from the various AI platforms.

The next stage of this study involved the development of a scoring system for DR screening. This scoring system aimed to assist healthcare professionals in considering the diverse risk factors associated with the development of DR. The scoring system was based on the responses provided by the clinician and the outputs generated by different AI platforms in response to various clinical case scenarios. Six risk factors were identified from the clinical case scenarios used in the questionnaire that appeared to be relevant in determining the right timing for DR screening. These include: (1) the patients’ age; (2) the type of diabetes; (3) diabetes control; (4) the presence of concurrent systemic conditions such as obesity, high BMI, renal disease, hypertension, dyslipidaemia, and tobacco use; (5) familial predisposition to diabetes; and (6) pregnancy status. The identification of risk factors that require prompt screening were assigned a score of three points for each risk factor based on the urgency of requiring DR screening. Risk factors that require screening within a year were assigned two points, while risk factors that require screening within five years were assigned one point for each risk factor. In the absence of a risk factor, a score of 0 would be assigned to it. The computation of a cumulative DR screening score would be conducted, followed by the provision of a categorical classification for the timing of screening based on the DR screening scores. The DR scores were classified into three groups according to the range of scores obtained during a specific timing for DR screening.

Considering the nature of the study, the study was exempted from institutional review board.

### Statistical analysis

The inter-rater reliability agreements between the different clinicians, different AI platforms, and the ‘majority clinician response’ and ‘majority AI response’ were calculated on DATAtab: Online Statistics Calculator (DATAtab e.U. Graz, Austria. URL https://datatab.net) using Fleiss Kappa and Cohen’s Kappa analysis. The Kappa result is interpreted as follows: ĸ values ≤ 0 as indicating no agreement and 0.01–0.20 as none to slight, 0.21–0.40 as fair, 0.41– 0.60 as moderate, 0.61–0.80 as substantial, and 0.81–1.00 as almost perfect agreement [22].

## Results

In the first phase of the study, the inter-rater reliability calculated by the Fleiss kappa test showed that there was a fair agreement between the 5 clinicians with κ = 0.25. The Fleiss Kappa showed that there was a fair agreement between ChatGPT 3.5, ChatGPT 4.0 and Bing AI with κ = 0.29. There was complete agreement between the ‘majority clinician response’ and the ‘majority AI response’ in 45% (n = 9) of the real-life clinical case scenarios. The Cohen’s Kappa showed that there was a fair agreement between ‘majority clinician response’ and ‘majority AI response’ with κ = 0.32. The inter-rate reliability agreements between the individual AI platforms and the ‘majority clinician response’ for ChatGPT 3.5v, ChatGPT 4.0v, and Bing AI were 0.24, 0.37, and 0.25, respectively.

We noted six risk factors that appeared to be relevant in determining the right timing for DR screening based on clinicians’ and different AI platforms’ responses to a set of 20 hypothetical AI-generated real-life clinical case scenarios. Individuals with poorly controlled or uncontrolled diabetes, as well as those with systemic co-morbidities, required prompt screening and were thus assigned a score of 3 points for each risk factor. Individuals with a family history of diabetes and pregnant women with diabetes were required to be screened within a year and were given two points for each risk factor. Additionally, individuals without the aforementioned risk factors required screening within the first five years of DM diagnosis. The patient’s age and type of diabetes had little influence on the need for immediate or early screening strategies for DR. As a result, patients over the age of 45 or with type 2 diabetes were assigned a score of one for each criterion, whereas patients under the age of 45 or with type 1 diabetes were not assigned any points. The DR screening score ranged from 0 to 10 for each clinical case scenario in this study, and three categories were formed based on these DR screening scores: (a) scores between 0 and 2 for cases requiring screening within 5 years, (b) scores between 3 and 5 for cases requiring screening within 1 year, and (c) scores between 6 and 12 for cases requiring immediate screening (Table 1).

**Table 1:** Evaluation of risk factors and calculation of diabetic retinopathy screening scores for individual clinical case scenarios

Case No.	Age	Type of DM	DM control	Systemic co-morbidities	Family History of DM	Pregnancy	Majority Response	DR Screening Score
1	46	2	Poor	Yes	No	No	Immediate	8
2	30	1	Good	No	Yes	No	Within 5 years	2
3	55	2	Poor	Yes	No	No	Immediate	8
4	28	2	Good	No	Yes	Yes	Within 1 year	5
5	50	2	Poor	Yes	Yes	No	Immediate	10
6	35	2	Good	No	No	No	Within 5 years	1
7	60	2	Poor	Yes	Yes	No	Immediate	10
8	40	2	Good	Yes	Yes	Yes	Immediate	8
9	48	2	Poor	Yes	No	No	Immediate	8
10	25	1	Good	No	No	No	Within 5 years	0
11	55	2	Poor	Yes	Yes	No	Immediate	10
12	33	2	Good	No	No	Yes	Within 1 year	3
13	52	2	Poor	Yes	No	No	Immediate	8
14	38	1	Good	No	Yes	No	Within 5 years	2
15	58	2	Poor	Yes	Yes	No	Immediate	10
16	29	1	Poor	No	Yes	Yes	Immediate	7
17	46	2	Poor	Yes	No	No	Immediate	8
18	22	1	Good	No	No	No	Within 5 years	0
19	56	2	Poor	Yes	Yes	No	Immediate	10
20	31	2	Good	No	No	Yes	Within 1 year	3

Abbreviations: DM– diabetes mellitus; DR– diabetic retinopathy

## Discussion

With the support of AI and clinicians, this one-of-a-kind study identifies risk factors of varying significance that may be important and relevant in determining the timing of DR screening in a newly diagnosed case of DM. The study also includes a screening score that may help non-ophthalmologists and even ophthalmologists from other specialties decide when to refer patients for DR screening to a trained retina specialist.

The prevalence of DM and, consequently, DR, as well as the availability of medical personnel and retina imaging tools for screening, differ by geographic area [7]. A number of risk factors influence the DR screening of newly diagnosed diabetic cases, which are either independent or interdependent on one another. The American Diabetes Association’s (ADA) recommendations for DR screening in newly diagnosed DM cases are the most widely accepted guidelines worldwide. According to the ADA, screening recommendations for DM patients were primarily based on two risk factors: type of DM and pregnancy status [5]. Community-based studies have identified over 12 risk factors that can hasten the development or progression of DR over time [23]. Therefore, the ADA guidelines appear to be overly simplified and inadequate. Also, it is not always possible for a retina specialist to inquire about and diagnose these risk factors using various laboratory tests in real-time situations. As a result, developing a strategy and scoring system based on a few key risk factors that is acceptable and routinely followed in clinical practice is becoming increasingly important. Individual national screening strategies have been developed to determine the timing of retina screening, the personnel who will conduct the screening, and the manner in which the screening must be performed based on disease prevalence and other risk factors [10]. These recommendations are intended to serve as a guide for ophthalmologists rather than for referring DM specialists. There is no uniform strategy for DR screening, even among retina specialists. Even in the current study, we found only a moderate level of agreement among retina specialists. In order to address this, we used the ‘majority clinician response,’ i.e., the best response was chosen as the most preferred timing for screening, establishing the most preferred practice pattern followed by clinicians.

Several latest generation chatbots developed using LLM-based generative AI applications have demonstrated promising results in generalizing to previously unseen tasks, including medical question-answering requiring scientific expert knowledge [24–26]. In order to formulate an answer, LLM understands the medical context, recall, and interpret relevant medical information and produces a response in a text-based format. Although reported performance in ophthalmology has been mixed, LLM appear to have potential for use in eye health care applications. LLM-based generative AI with ChatGPT and ChatGPT 4.0v has been used in retina for a variety of indications, including International Coding of Diseases (ICD) for various case encounters [27, 28]. AI’s current role in DR is limited to preventive care, i.e., screening [14, 17]. According to the ADA, AI can be used as an alternative to traditional screening methods in DR [29]. AI’s current role in DR is to screen retinal images for the presence or absence of DR or sight-threatening DR [30]. However, AI should not be used in patients who have known DR, have received prior DR treatment, or have symptoms of vision impairment. Different chatbot applications respond to the same situations in different ways [31]. Even in the current study, the different AI platforms only agreed on a moderate level for the same clinical case scenario. To address this issue, the ‘majority AI response’ was selected as the most preferred time for DR screening based on AI. In order to improve both the precision and speed of responses, the AI platform must receive the most up-to-date information and have real-time access to the internet. In this study, we observed that ChatGPT 4.0v performed better and had closer agreements with clinician responses than the other two AI platforms.

DR is a retinal complication of prolonged DM that affects the retinal microvasculature [32]. As a result, the longer the duration of DM, the higher the risk of developing DR or sight-threatening DR is usually considered. Individual national screening guidelines for DR, as well as global guidelines developed by the International Council of Ophthalmology, have identified uncontrolled DM and presence of hypertension and other systemic co-morbidities as risk factors which could alter the course of DR [10]. This study identified six factors based on clinicians’ and AI’s responses. According to the findings of this study, patients with poorly controlled blood sugar levels or those with co-existing systemic co-morbidities required immediate DR screening, whereas those with a family history of DM or diabetic patients who were pregnant preferred to be screened within a year. The absence of any of these risk factors made these cases less urgent for screening, and they were screened over a 5-year period. The ADA guidelines instruct patients with type 2 diabetes to undergo screening immediately after diagnosis because many patients with type 2 diabetes have the disease for a long time before being diagnosed, and immediate screening is therefore recommended. Patients with type 1 diabetes, on the other hand, must be screened within 5 years of disease diagnosis [5]. Based on this, we assigned one point to each risk factor, such as patients over the age of 45 and type 2 diabetes. In this study, we discovered that the patient’s age and type of diabetes had less of an impact on the timing of DR screening in the absence of other risk factors such as poor DM control, co-existing systemic comorbidities, pregnancy, and a family history of DM. As a result, this study identified risk factors of varying significance that may influence the progression of DR and, as a result, the timing of screening in newly diagnosed cases of DM.

The study has some limitations. The number of clinical case scenarios generated hypothetically could have been increased, or the clinical case scenarios could have been presented to a larger number of clinicians and AI applications. The study placed little emphasis on comparing the responses of various AI applications. Another limitation of this study was that when developing the case scenarios, the race of the patient was not taken into account, and the questions were only presented to Indian ophthalmologists. Instead, the clinical case scenarios could have been presented to international ophthalmologists, increasing the study’s global acceptability. The term ‘majority’ clinician and AI responses used in this study may mislead readers and be misinterpreted as the best response from the entire field of retina specialists and AI platforms. The recommendations made in this study are based on expert opinions and clinically unvalidated LLMs that have not consistently performed well in ophthalmologic field expertise in many other studies. Furthermore, inter-rater agreement was at the best only moderate between LLMs and ophthalmologists, as well as between ophthalmologists themselves. As a result, without proper clinical validations, the study recommendations may be true and beneficial to some patients but not to all. Nonetheless, the study has the advantage of developing a simplified screening strategy for non-ophthalmologists to send newly detected diabetic cases for retina screening by combining real-world clinical experience with up-to-date AI information. After validating these screening recommendations, it is possible that they will be integrated into the hospital’s electronic medical record system, alerting ophthalmologists and non-ophthalmologists to refer patients with newly detected DM for timely DR screening and thus helping to reduce the number of unnecessary referrals to retina specialists who are less likely to have any form of DR.

In conclusion, AI has the potential to be highly influential in the screening of DR in newly diagnosed patients with DM by creating an innovative DR screening score. Further studies are necessary for validating the DR screening score prior to its application as a guide in practical clinical scenarios.

### Electronic supplementary material

Below is the link to the electronic supplementary material.

Supplementary Material 1: Supply Clinical case scenarios generated by ChatGPT 3.5v

## Data Availability

The datasets used and/or analysed during the current study are available from the corresponding author on reasonable request.

## Declarations

### Research involving animal participants

“This article does not contain any studies with animals performed by any of the authors.”

## Abbreviations

DM: diabetes mellitus
DR: diabetic retinopathy
AI: artificial intelligence
LLM: large language model
ADA: American Diabetes Association
