# Supplementary material for: Recommendations for initial diabetic retinopathy screening of diabetic patients using large language model-based artificial intelligence in real-life case scenarios
Source: Int J Retina Vitreous. 2024 Jan 24;10:11. doi: 10.1186/s40942-024-00533-9 (PMC10809735; doi:10.1186/s40942-024-00533-9)
Supplement: Supplementary file 1 — Supplementary Material 1: Supply Clinical case scenarios generated by ChatGPT 3.5v [file 40942_2024_533_MOESM1_ESM.docx]

***Clinical case scenarios generated by ChatGPT 3.5v***

1. 46-year-old male, poorly controlled Type 2 diabetes, hypertension, obesity
2. 30-year-old female, well-controlled Type 1 diabetes, family history of diabetes
3. 55-year-old male, poorly controlled Type 2 diabetes, history of smoking, high cholesterol, diabetic kidney disease
4. 28-year-old pregnant female, well-managed diabetes, family history of diabetes
5. 50-year-old male, moderately controlled Type 2 diabetes, high blood pressure, obesity, family history of diabetes
6. 35-year-old female, well-controlled Type 1 diabetes, no family history of diabetes, normal body mass index (BMI)
7. 60-year-old male, poorly controlled Type 2 diabetes, high blood pressure, history of smoking, obesity
8. 40-year-old pregnant female, well-managed diabetes, obese, family history of diabetes
9. 48-year-old male, poorly controlled Type 2 diabetes, diabetic kidney disease, high cholesterol, obesity
10. 25-year-old female, well-controlled Type 1 diabetes, no family history of diabetes, healthy BMI
11. 55-year-old male, poorly controlled Type 2 diabetes, high blood pressure, obesity, family history of diabetes
12. 33-year-old pregnant female, well-managed diabetes, no family history of diabetes, normal BMI
13. 52-year-old male, poorly controlled Type 2 diabetes, diabetic kidney disease, high cholesterol, obesity
14. 38-year-old female, well-controlled Type 1 diabetes, no family history of diabetes, normal BMI
15. 58-year-old male, poorly controlled Type 2 diabetes, high blood pressure, obesity, family history of diabetes
16. 29-year-old pregnant female, poorly-managed diabetes, family history of diabetes
17. 46-year-old male, poorly controlled Type 2 diabetes, diabetic kidney disease, high cholesterol, obesity
18. 22-year-old female, well-controlled Type 1 diabetes, no family history of diabetes, healthy BMI
19. 56-year-old male, poorly controlled Type 2 diabetes, high blood pressure, obesity, family history of diabetes
20. 31-year-old pregnant female, well-managed diabetes, no family history of diabetes, normal BMI
